# Nuclear localization of the dehydrin OpsDHN1 is determined by histidine-rich motif

**DOI:** 10.3389/fpls.2015.00702

**Published:** 2015-09-07

**Authors:** Itzell E. Hernández-Sánchez, Israel Maruri-López, Alejandro Ferrando, Juan Carbonell, Steffen P. Graether, Juan F. Jiménez-Bremont

**Affiliations:** ^1^Laboratorio de Biología Molecular de Hongos y Plantas, División de Biología Molecular, Instituto Potosino de Investigación Científica y Tecnológica ACSan Luis Potosí, México; ^2^Instituto de Biología Molecular y Celular de Plantas, Universidad Politécnica de Valencia-Consejo Superior de Investigaciones CientíficasValencia, Spain; ^3^Department of Molecular and Cellular Biology, University of GuelphGuelph, ON, Canada

**Keywords:** dehydrin, BiFC, homodimer, histidine-rich motif, nuclear/cytoplasmic localization

## Abstract

The cactus OpsDHN1 dehydrin belongs to a large family of disordered and highly hydrophilic proteins known as Late Embryogenesis Abundant (LEA) proteins, which accumulate during the late stages of embryogenesis and in response to abiotic stresses. Herein, we present the *in vivo* OpsDHN1 subcellular localization by N-terminal GFP translational fusion; our results revealed a cytoplasmic and nuclear localization of the GFP::OpsDHN1 protein in *Nicotiana benthamiana* epidermal cells. In addition, dimer assembly of OpsDHN1 *in planta* using a Bimolecular Fluorescence Complementation (BiFC) approach was demonstrated. In order to understand the *in vivo* role of the histidine-rich motif, the OpsDHN1-ΔHis version was produced and assayed for its subcellular localization and dimer capability by GFP fusion and BiFC assays, respectively. We found that deletion of the OpsDHN1 histidine-rich motif restricted its localization to cytoplasm, but did not affect dimer formation. In addition, the deletion of the S-segment in the OpsDHN1 protein affected its nuclear localization. Our data suggest that the deletion of histidine-rich motif and S-segment show similar effects, preventing OpsDHN1 from getting into the nucleus. Based on these results, the histidine-rich motif is proposed as a targeting element for OpsDHN1 nuclear localization.

## Introduction

Dehydrins (DHNs) belong to a large family of highly hydrophilic proteins known as Late Embryogenesis Abundant (LEA) proteins. Proteins in the LEA group accumulated in response to stress conditions that cause cellular dehydration, such as low temperatures, high salinity, and drought (Hanin et al., 2011). Based on *in vitro* experiments, various functions have been proposed for DHNs, for example as cryoprotectants, chaperones, antioxidants, and ion sequestrants (Hara, 2010). Transgenic plants that overexpress DHN genes in diverse plant species are associated with drought, cold, salinity, and heavy metal tolerance. Intriguingly, DHNs are intrinsically disordered proteins (IDPs) which prevents them from denaturing during desiccation or at freezing temperatures (Tompa and Kovacs, 2010; Graether and Boddington, 2014; Hernández-Sánchez et al., 2014). Even though the DHNs are the most studied group of LEAs, the precise molecular mechanisms by which these proteins exert their function are still unknown.

Strictly speaking, DHNs contain at least one motif, named the K-segment (EKKGIMDKIKEKLPG), which can interact with macromolecules and specific membrane regions to protect them from stress damage (Koag et al., 2009). On the other hand, DHNs may contain other segments, such as the S-segment (5–7 serine residues). Phosphorylation of this motif has been correlated with nuclear localization of the protein; however, some DHNs exhibit nuclear localization independently of the phosphorylation state of the S-segment, even DHNs which does not possess an S-segment have been found in the nucleus (Riera et al., 2004; Rorat, 2006). The Y-segment (T/VDEYGNP), located near the N-terminus, shares a certain degree of identity with a portion of the nucleotide-binding site of plant and bacterial chaperones (Close, 1996). Besides these common segments, the presence of histidine-rich motifs (H-X_3_-H, HH, and H_*n*_) have been reported to be involved in the formation of complexes with metal ions, and act as ion chelators and as DNA binding sites (Hara et al., 2001, 2005; Hara, 2010).

DHNs are ubiquitous plant proteins localized in different cell compartments, such as the nucleus, chloroplasts, vacuole, rough endoplasmic reticulum, mitochondria, cytoplasm, and membranes (Heyen et al., 2002; Mueller et al., 2003; Carjuzaa et al., 2008). In addition, DHNs accumulate in different tissues during plant growth and development, and in response to stress (Nylander et al., 2001; Rorat, 2006). We previously isolated the cold-inducible *OpsDHN1* dehydrin from a cDNA library of *Opuntia streptacantha* (Ochoa-Alfaro et al., 2012). The *OpsDHN1* gene encodes an acidic SK_3_ DHN that contains an intron located within the sequence that itself encodes the S-segment; this element is conserved in phase and location in most SK_n_-type DHNs (Jiménez-Bremont et al., 2013). Transgenic Arabidopsis plants overexpressing *OpsDHN1* gene display freezing tolerance, suggesting that OpsDHN1 participates in cold stress responsiveness (Ochoa-Alfaro et al., 2012). Recently, the OpsDHN1 homodimer formation using the split-ubiquitin yeast two-hybrid (Y2H) system and by size-exclusion chromatography of the recombinant protein was detected (Hernández-Sánchez et al., 2014).

Herein, we show the *in vivo* subcellular localization of OpsDHN1 protein by the translational fusion with GFP in *Nicotiana benthamiana* leaves. Furthermore, the Bimolecular Fluorescence Complementation (BiFC) assay allowed direct visualization *in planta* of OpsDHN1 dimer formation and its subcellular localization. In this regard, deletion of the regions that contain the histidine- and serine-rich motifs in OpsDHN1 protein affected its subcellular localization. Our findings constitute the first report about the *in vivo* DHN-DHN interaction and disclose relevant sequences for the nuclear localization of the protein.

## Materials and methods

### Plant material

Seeds of *Nicotiana benthamiana* were sown on a mix of vermiculite and soil (1:1), and grown for 3–4 weeks in controlled greenhouse conditions under long day photoperiod cycles (16 h light/8 h dark) at 22°C ± 1°C.

### Vector construction

The *OpsDHN1* open reading frame was amplified by PCR using Phusion high-fidelity DNA polymerase (Thermo scientific, Carlsbad, CA, USA). The OpsDHN1-ΔHis and OpsDHN1-ΔS versions were generated by fusion of two *OpsDHN1* High-fidelity PCR fragments, comprising bases 1-333/415-747 and 1-201/249-747, respectively. To fuse the PCR products, *Kpn*I restriction sites were included in each primer sequence. Subsequently, these products were digested with *Kpn*I enzyme (Invitrogen, Carlsbad, CA, USA) and ligated using T4 DNA ligase (Invitrogen).

The OpsDHN1, OpsDHN1-ΔHis, and OpsDHN1-ΔS versions were cloned into the pCR8/GW/TOPO (Invitrogen) entry vector. For the subcellular localization analyses, each entry vector was sub-cloned into the pMDC43 binary vector (Curtis and Grossniklaus, 2003). To perform Bimolecular Fluorescence Complementation (BiFC) experiments each entry construct was sub-cloned into the pYFN43 and pYFC43 binary vectors (Belda-Palazón et al., 2012). The sub-cloning was performed by site-specific recombination using Gateway LR Clonase II Enzyme Mix (Invitrogen). These vectors were introduced into *Agrobacterium tumefaciens* GV3101/pMP90 strain.

### Plant transient transformation

The *N. benthamiana* leaves were agro-infiltrated with GV3101/pMP90 cells carrying the appropriate plasmid combinations. In order to suppress gene silencing, *A. tumefaciens* cells that express the tomato bushy stunt virus p19 protein (Voinnet et al., 2003) from Plant Bioscience Limited (PBL, Norwich, UK) were used in the co-infiltration method, as previously reported by Belda-Palazón et al. (2012). Briefly, the *A. tumefaciens* cells grown to about an OD_600_ of 2.0 were collected and re-suspended in a similar volume of infiltration buffer (10 mM MgCl_2_, 10 mM MES pH 5.6, 200 mM acetosyringone). The strains were incubated at 28°C for 3 h. An equal mixture of *Agrobacterium* strains containing the appropriate translational fusion constructs and the p19 plasmid was prepared for co-infiltration into the abaxial air space of *N. benthamiana* leaves with a needleless syringe; at least two transformed leaves of three plants of similar age were assayed for fluorescence under a confocal microscope 3 days post-infiltration. The experiments were repeated at least three times for each construct.

### Nuclei staining

The reagent 4′,6-diamidino-2-phenylindole (DAPI; Sigma, St. Louis, MO) was used for staining nuclei. Before fluorescence confocal microscopy analysis the agro-infiltrated *N. benthamiana* leaves were removed from the plant and cut into one-inch diameter circles; these were incubated in distilled water supplemented with 5 μg/ml DAPI for 30 min. Afterwards, leaf sections were mounted on a microscope slide and covered with distilled water for observation through the leaf abaxial side.

### Fluorescence confocal microscopy

Confocal imaging was carried out using an inverted confocal laser-scanning microscope (LSM 780, Carl Zeiss, Jena, Germany). The laser excitation wavelength was 488 nm and the spectral detection was set between 497 and 537 nm for GFP and 684–758 nm for chlorophyll fluorescence, using the beam splitter MBS 488. For DAPI laser excitation the wavelength was set to 405 nm and detection was made at 410–492 nm. The objective used was a C-Apochromat 10x and 40x/1.20W. Image analysis was carried out with the ZEN imaging software (Carl Zeiss).

### Analysis of OpsDHN1 sequence

The putative nuclear localization of OpsDHN1 protein was analyzed using the YLoc web server (http://abi.inf.uni-tuebingen.de/Services/YLoc/webloc.cgi) (Briesemeister et al., 2010). The nuclear localization signals (NLS) in OpsDHN1 protein were determined by cNLS mapper program with a cut-off score of 5 (http://nls-mapper.iab.keio.ac.jp/cgi-bin/NLS_Mapper_form.cgi) (Kosugi et al., 2009). Homology searches were conducted using the BLAST program (BLASTP) in non-redundant GenBank database of the National Center of Biotechnology Information (NCBI; http://www.ncbi.nlm.nih.gov). The protein sequence alignment was carried out using the T-Coffee program at the EBI website (http://www.ebi.ac.uk/).

## Results

### Subcellular localization of OpsDHN1 dehydrin

**Figure 1:**
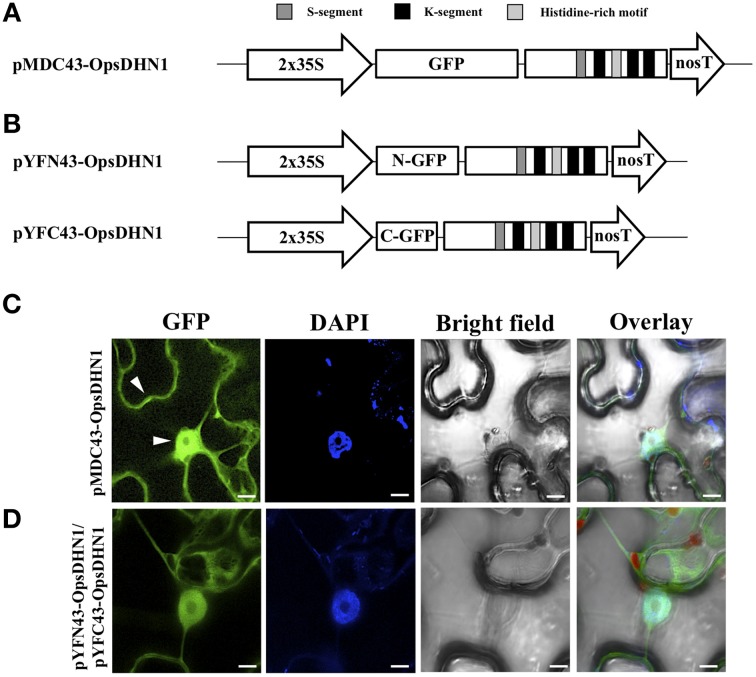
*****In vivo*** OpsDHN1 subcellular localization and dimer formation. (A)** Schematic representation of the pMDC43-OpsDHN1 construct. **(B)** Schematic representation of BiFC pYFN43- and pYFC43-OpsDHN1 vectors. **(C)** Cytoplasm and nuclear localization of the GFP::OpsDHN1 translational fusion in *N. benthamiana* epidermal cells. White arrowheads indicate cytosolic and nuclear signals. **(D)** Detection of the OpsDHN1 dimer formation by BiFC approach. The fluorescence was assessed by laser-scanning confocal microscopy. From left to right: the GFP and DAPI fluorescence spectrum, bright field, chlorophyll fluorescence and overlay signals. The S-segment, K-segments, and histidine-rich motif are depicted as dark-gray, black, and light-gray boxes, respectively. The scale bar corresponds to 10 μm.

In order to determine the *in vivo* subcellular localization of the OpsDHN1 protein, we generated an N-terminal translational fusion with GFP (Figure 1A). The *OpsDHN1* open reading frame was cloned into the pCR8 entry vector and sub-cloned into the pMDC43 gateway binary vector (Curtis and Grossniklaus, 2003). The 35S:GFP::OpsDHN1 construct was analyzed by a transient expression system in agro-infiltrated *Nicotiana benthamiana* leaves. The fluorescence was observed through a laser-scanning confocal microscope. DAPI staining was used to visualize nuclei localization. As shown in Figure 1C, the OpsDHN1 protein shows a dual cytoplasmic and nuclear localization in tobacco epidermal cells.

### *In planta* OpsDHN1-OpsDHN1 protein interaction

Previously, we reported the OpsDHN1-OpsDHN1 protein interaction by the split-ubiquitin yeast two-hybrid (Y2H) assay (Hernández-Sánchez et al., 2014). In order to demonstrate that this dimerization also occurs *in planta*, a Bimolecular Fluorescence Complementation (BiFC) analysis was performed. For this experiment, the pCR8-OpsDHN1 entry vector was sub-cloned into the pYFN43 and pYFC43 BiFC gateway binary vectors (Belda-Palazón et al., 2012) (Figure 1B); both constructs were co-infiltrated in *N. benthamiana* leaves. The GFP fluorescence complementation was analyzed by laser-scanning confocal microscope, and the nuclei were DAPI stained. As a positive BiFC interaction controls, we included the interaction between two subunits of the Arabidopsis SnRK kinase, AKIN10 and AKINβ2 (Ferrando et al., 2001), which showed a fluorescence signal under confocal microscope (Supplementary Figure S1). To verify that there is no self-activation of the pYFN43-OpsDHN1 construct, this vector was agro-infiltrated in *N. benthamiana* leaves (Supplementary Figure S1). In addition, the interaction between OpsDHN1 and AKINβ2 proteins (pYFN43-OpsDHN1/pYFC43-AKINβ2) was included as a negative interaction control (Supplementary Figure S1). Our BiFC results show that the OpsDHN1 protein is able to interact with itself in plant cells (Figure 1D), which is consistent with the split-ubiquitin Y2H data (Hernández-Sánchez et al., 2014). One can observe that the homodimer is located in both the cytoplasm and nucleus of tobacco epidermal cells (see Figure 1D).

### The histidine-rich motif is required for OpsDHN1 nuclear localization

**Figure 2:**
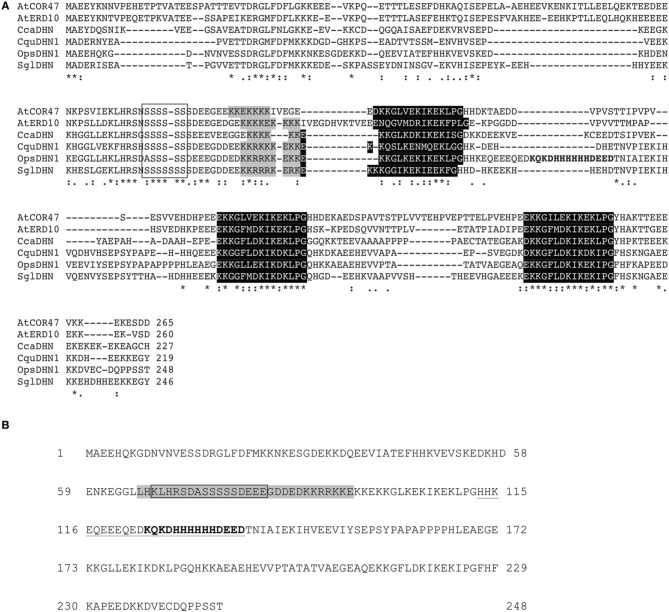
**Multiple sequence alignment of SK_3_ DHN proteins and the OpsDHN1**. **(A)** Identical residues (asterisk) in the six DHN sequences and conserved amino acid substitutions (dots) are indicated, dashes show gaps in the amino acid sequences introduced to optimize alignment, conserved regions, and distinctive motifs of the group: S-segment (open box), poly-lysine rich sequence (gray shadow), K-segments (black shadow), and particular histidine-rich motif of OpsDHN1 (bold letters). **(B)** Schematic representation of bipartite NLS (gray shadow) and the metal-binding site (bold letters) in OpsDHN1 protein sequence, deleted regions: in OpsDHN1-ΔSer version the open box and for OpsDHN1-ΔHis version the underlined sequence.

The dehydrin (DHN) family is described in terms of three conserved motifs: K, S, and Y-segments (Close, 1996). In the multiple alignment of the DHNs amino acid sequences, we showed that the OpsDHN1 displays a particular histidine tract (H_6_) in comparison to other SKn-type DHNs from *Chenopodium quinoa* (AGM15308), *Coffea canephora* (ABC68275), *Suaeda glauca* (AEA29617), and *Arabidopsis thaliana* [AtCOR47 (At1g20440), AtERD10 (At1g20450)] (Figure 2A).

**Figure 3:**
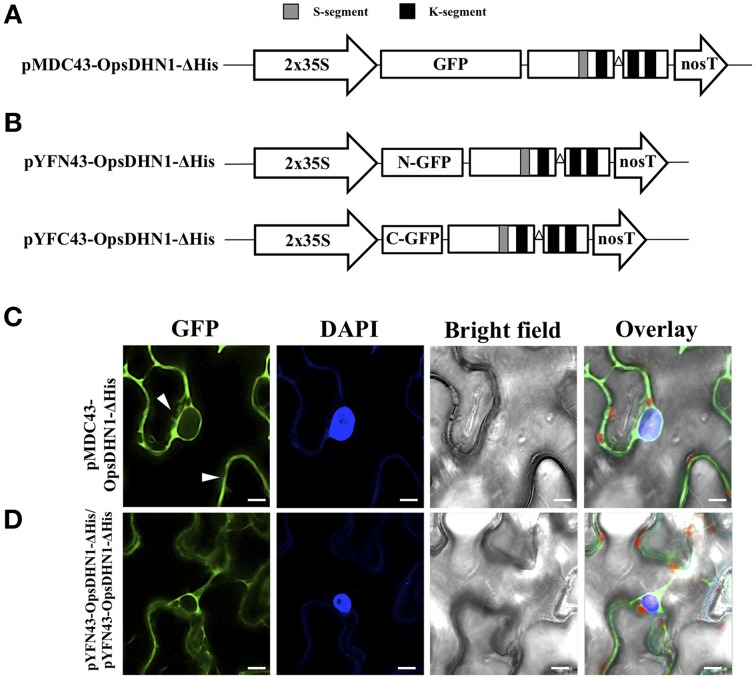
**The OpsDHN1 histidine-rich motif is required for its nuclear subcellular localization. (A)** Schematic representation of the pMDC43-OpsDHN1-ΔHis construct. **(B)** Schematic representation of BiFC pYFN43- and pYFC43- OpsDHN1-ΔHis vectors. **(C)** Cytoplasm localization of GFP::OpsDHN1-ΔHis translational fusion in *N. benthamiana* leaves. White arrowheads indicate cytosolic and nuclear compartments. **(D)** BiFC assay of OpsDHN1-ΔHis version. Transient fluorescent expression was analyzed by laser-scanning confocal microscopy. From left to right: the GFP and DAPI fluorescence spectrum, bright field, chlorophyll fluorescence and overlay signals. The S-segment and K-segments are depicted as dark-gray box and black boxes, respectively. The deleted histidine-rich motif is represented by an open triangle. The scale bar corresponds to 10 μm.

Previously, we found that deletion of a region that comprises this motif (Figure 2B) affected dimer formation in split-ubiquitin Y2H system (Hernández-Sánchez et al., 2014). In order to determine whether the histidine-rich motif of the OpsDHN1 protein plays a role *in planta* dimer formation and subcellular localization, we generated the OpsDHN1-ΔHis deleted version, which lacks the central histidine-rich motif (HHKEQEEEQEDKQKDHHHHHHDEED; Figure 2B) that is located between the first and second K-segment. This construct was assayed for subcellular location by N-terminal translational fusion with GFP. The OpsDHN1-ΔHis version was cloned into pCR8 entry vector and sub-cloned into the pMDC43 gateway binary vector (Figure 3A). The 35S:GFP::OpsDHN1-ΔHis construct was analyzed by a transient expression system in agro-infiltrated *N. benthamiana* leaves. The fluorescence was visualized through a laser-scanning confocal microscope (Figure 3C). Staining with DAPI was used to visualize nuclei localization. Our data reveal restricted cytoplasm localization for OpsDHN1-ΔHis construct, excluding the GFP signal from the nuclei of *N. benthamiana* epidermal cells (Figure 3C).

**Figure 4:**
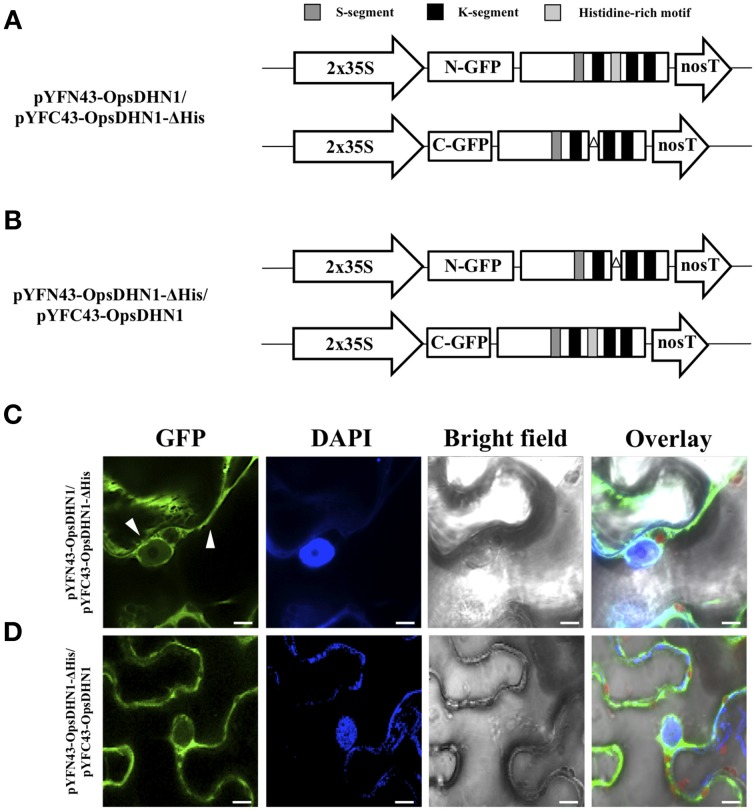
**Visualization of OpsDHN1/OpsDHN1-ΔHis dimer formation using BiFC approach**. **(A,B)** Schematic representation of OpsDHN1 derived versions cloned into pYFN43 and pYFC43 vectors. **(C,D)** BiFC analysis of OpsDHN1/OpsDHN1-ΔHis and swapped constructs. White arrowheads indicate cytosolic and nuclear compartments. The fluorescence was assessed by laser-scanning confocal microscopy. From left to right: the GFP and DAPI fluorescence spectrum, bright field, chlorophyll fluorescence and overlay signals. The S-segment, K-segments, and histidine-rich motif are depicted as dark-gray, black, and light-gray boxes, respectively. The deleted histidine-rich motif is represented by open triangle. The scale bar corresponds to 10 μm.

In addition, we analyzed the ability of OpsDHN1-ΔHis to form dimers *in vivo.* The pCR8-OpsDHN1-ΔHis construct was sub-cloned into both BiFC gateway binary vectors (Figure 3B). The positive BiFC interaction control between AKIN10 and AKINβ2 was included; in the same way, the pYFN43-OpsDHN1-ΔHis vector and the interaction between pYFN43-OpsDHN1/pYFC43-AKINβ2 constructions were included as a non-auto fluorescent and negative interaction controls, respectively (Supplementary Figure S1). As shown in Figure 3D, the OpsDHN1-ΔHis version is able to assembly dimers in the *N. benthamiana* cytoplasm. Moreover, we evaluated whether the deletion of the histidine-rich motif in one copy of OpsDHN1 affects its interaction and localization with the full-length version. Co-expression of OpsDHN1/OpsDHN1-ΔHis or OpsDHN1-ΔHis/OpsDHN1 constructs (Figures 4A,B) showed restricted cytoplasm localization in *N. benthamiana* epidermal cells for the dimer (Figures 4C,D). With these experiments, we obtained the same result as when the OpsDHN1-ΔHis/OpsDHN1-ΔHis interaction was tested.

### The S-segment is also implicated in OpsDHN1 nuclear localization

**Figure 5:**
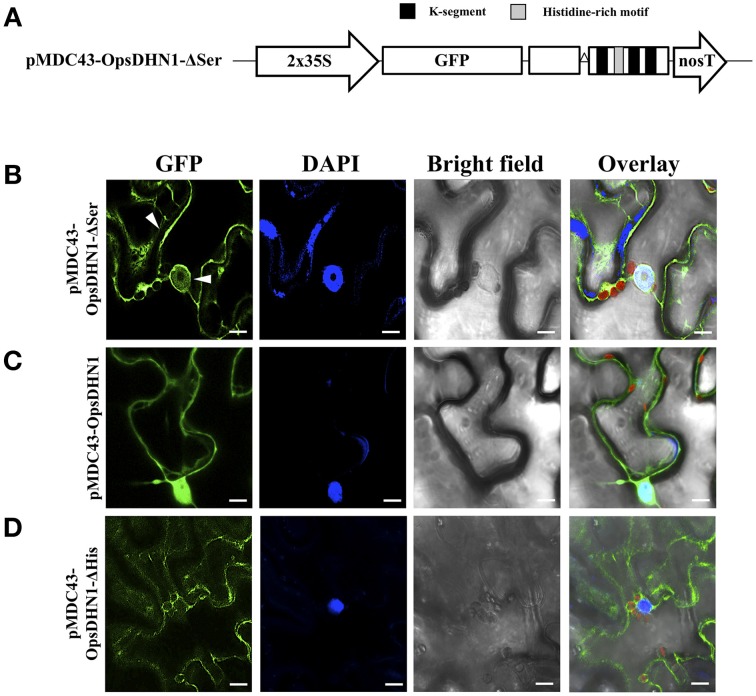
**The OpsDHN1 S-segment is involved in its nuclear location. (A)** Schematic representation of the pMDC43-OpsDHN1-ΔSer construct. Fluorescent visualization of **(B)** GFP::OpsDHN1-ΔSer, **(C)** GFP::OpsDHN1, **(D)** GFP::OpsDHN1-ΔHis translational fusions in *N. benthamiana* leaves. White arrowheads indicate cytosol and nuclear signals. The fluorescence was examined by laser-scanning confocal microscopy. From left to right: the GFP and DAPI fluorescence spectrum, bright field, chlorophyll fluorescence and overlay signals. The K-segments and histidine-rich motif are depicted as black and light-gray boxes, respectively. The deleted S-segment is represented by open triangle. The scale bar corresponds to 10 μm.

The phosphorylation of DHN S-segment has been shown to play a role in its nuclear import (Riera et al., 2004). An analysis of the S-segment in DHNs reveals that several residues N-terminal and C-terminal to the 5–6 serine residues are also conserved. To analyze the *in vivo* putative role in the nuclear localization of the DHN S-segment, we generated the OpsDHN1-ΔSer version, in which the serine tract (KLHRSDASSSSSDEEE; see Figure 2B) was deleted. This construct was assayed for subcellular localization by N-terminal translational fusion with GFP (Figure 5A). For this aim, the OpsDHN1-ΔSer construct was cloned and sub-cloned into the pCR8 entry vector and pMDC43 binary vector, respectively. The fluorescent signal of the 35S:GFP::OpsDHN1-ΔSer construct was analyzed through a transient expression system in agro-infiltrated *N. benthamiana* leaves. The fluorescence was observed through a laser-scanning confocal microscope. DAPI staining was used to visualize nuclei localization. We found that the *N. benthamiana* epidermal cells exhibited strong fluorescence in the cytoplasm; however, there is a slight fluorescence remaining in the nuclei (Figure 5B), compared to the nuclear signal of 35S:GFP::OpsDHN1 (Figure 5C, Supplementary Figure S2). Our results suggest that the S-segment of OpsDHN1 is important but not essential for nuclear targeting of the protein.

## Discussion

Dehydrins (DHNs) are a versatile group of proteins with multiple functions to assist or protect plant cell under stress conditions (Hanin et al., 2011). However, the molecular mechanisms by which these proteins exert their functions inside the cell are not fully known. The study of the subcellular localization of the DHNs could shed new insights about its role inside the cell and the molecular mechanism through which these proteins attain their functions. We evaluated here the subcellular localization of the cactus pear OpsDHN1 by using GFP translational fusion. Our confocal analysis reveals a dual cytoplasmic and nuclear location for the OpsDHN1 protein in *Nicotiana benthamiana* epidermal cells (Figure 1C). These data agree with our *in silico* analysis using the YLoc web server (Briesemeister et al., 2010), which predicted a 52.7 and 47.3% cytoplasm and nuclear localization probability for OpsDHN1. In this regard, several reports revealed that DHN proteins are localized in diverse cell compartments, such as mitochondria, vacuole, chloroplasts, and in the vicinity of the plasma membrane (reviewed in Graether and Boddington, 2014). However, the most prevalent locations for DHNs are in the cytoplasm and nucleus. This dual subcellular localization has been reported for several DHNs such as: maize Rab17 (YSK_2_; Jensen et al., 1998), wheat WCS120 (K_6_; Houde et al., 1995) peach PCA60 (Y_2_K_8_; Wisniewski et al., 1999), and *Medicago truncatula* MtCAS31 (Y_2_K_4_; Xie et al., 2012).

Previously, we demonstrated using the split-ubiquitin yeast two-hybrid (Y2H) system that OpsDHN1 is able to dimerize (Hernández-Sánchez et al., 2014). Here our BiFC approach demonstrated that *in planta* OpsDHN1 is able to form a homodimer with a dual cytoplasm and nuclear distribution. Although the dimerization of TsDHN-2 (Y_2_SK_2_) from *Thellungiella salsuginea* has been previously demonstrated *in vitro* (Rahman et al., 2013), our data are the first to report an *in vivo* DHN-DHN interaction.

It is know that histidine is a common residue in DHN proteins (Hara et al., 2005, 2009, 2013; Xu et al., 2008; Hara, 2010). Interestingly it has been reported that DHNs can bind *in vitro* to several ligands, such as DNA, RNA, and metal ions, through lysine- and histidine-rich motives (Hara et al., 2005, 2009). In particular, a metal binding site rich in histidine residues was identified in the OpsDHN1 sequence (Ochoa-Alfaro et al., 2012; see Figure 2). In the previous split-ubiquitin Y2H work, we found that the OpsDHN1 histidine-rich motif is implicated in its dimer interaction (Hernández-Sánchez et al., 2014). In the present research work, we analyzed the OpsDHN1-ΔHis version for dimer capability and protein localization. The fluorescence data indicate that the histidine-rich motif is a crucial domain for OpsDHN1 nuclear localization in *N. benthamiana* epidermal cells (Figure 3C). DHNs that possess the His-His and/or His–X_3_–His signatures, such as Arabidopsis Rab18 (Y_2_SK_2_; Nylander et al., 2001) and LTI30 (K_6_; Puhakainen et al., 2004), maize Rab17 (YSK_2_; Jensen et al., 1998), and tomato TAS14 (YSK_2_; Godoy et al., 1994), are preferentially located in the plant nuclei. Additionally, a histidine-rich motif mediates the nuclear localization of *Capsicum annuum* silencing CaDC1 protein (Hwang et al., 2014). Considering the report about the cytoplasmic and nuclear localization of GFP, it might generate some controversy about our OpsDHN1 subcellular localization data; however, here we demonstrated that the OpsDNH1 localization is not an artifact, since our results show that the OpsDHN1-ΔHis version retains the GFP translational fusion in the cytosol.

In contrast to the split-ubiquitin Y2H result, the OpsDHN1 protein is able to interact with itself in the absence of its histidine tract *in planta* (Figure 3D). The difference observed between both systems might be due to the split-ubiquitin method. In this system, the OpsDHN1 could present differences in protein conformation, since in the bait its N-terminus is fused to a small membrane anchor (the yeast endoplasmic reticulum protein Ost4), and at its C-terminus to a reporter cassette composed of the C-terminal half of ubiquitin (Cub) and a transcription factor; in contrast to BiFC system, in which the fusion of the GFP moiety is only at the N-terminal of OpsDHN1 and the translational fusions are not anchored to any organelle.

In both BiFC assays, OpsDHN1/OpsDHN1-ΔHis, and OpsDHN1-ΔHis/OpsDHN1-ΔHis showed that dimers were only localized in the cytosol of the tobacco epidermal cells (Figures 4C,D). These results confirm that the histidine-rich motif is necessary for OpsDHN1 nuclear localization. These data show that the homodimer formation in the nucleus requires the presence of both intact monomers, since in the co-expression of OpsDHN1 full version and OpsDHN1-ΔHis version, only the full version will be found in the nucleus.

Proteins are translocated from the cytoplasm to the nucleus to perform basic cellular process, or in response to developmental and environmental signals (Jensen et al., 1998). Ricardi et al. (2012) suggested that the tomato ASR1 (LEA 7 group) homodimer could be pre-formed in the cytoplasm before nuclear import, or alternatively that the monomers were translocated to the nucleus to be dimerized within that compartment. It is possible that the OpsDHN1 histidine-rich motif mediates nuclear targeting through induction of a specific protein structure that could be recognized for nuclear import independently of protein dimerization.

In addition, we evaluated the OpsDHN1 S-segment for its proposed role in the nuclear targeting of some DHNs (Xu et al., 2008). For this aim, the GFP::OpsDHN1-ΔSer version was assayed for protein subcellular localization in *N. benthamiana* epidermal cells. Our data show a slight nuclear fluorescent signal from the OpsDHN1-ΔSer construct (Figure 5), suggesting that S-segment may be relevant for OpsDHN1 nuclear localization but not essential. In the case of maize Rab17 and Arabidopsis ERD14 DHNs, it has been reported that the S-segment can undergo phosphorylation by casein kinase 2 (Plana et al., 1991; Alsheikh et al., 2003). Goday et al. (1994) reported that phosphorylation is important for Rab17's capacity to bind to nuclear localization signal (NLS) peptides *in vitro*. *In silico* analysis of OpsDHN1 using cNLS mapper (Kosugi et al., 2009) revealed a potential bipartite NLS that includes its S-segment (Figure 2B). This putative NLS signal comprises 30 amino acids (65–95 amino acid of OpsDHN1), while the serine segment covers 16 amino acids (67–83 amino acid of OpsDHN1). This could explain the low GFP signal observed in the nuclei for the OpsDHN1-ΔSer version, since the NLS may not have been eliminated.

In summary, we show that the OpsDHN1 protein has a cytoplasmic and nuclear localization *in planta* cells. BiFC data revealed that the OpsDHN1 homodimerization does occur *in vivo*. Our data suggest that OpsDHN1 localization is mediated by the histidine-rich motif and to some degree by the S-segment. However, more *in vivo* studies are necessary to understand the physiological role of these motifs present in OpsDHN1 sequence.

### Conflict of interest statement

The reviewer, Sabina Vidal, declares that, despite having co-edited the Research Topic “Dehydrins in plant stress responses” with the co-author Juan F. Jiménez-Bremont, the review process was conducted objectively. The authors declare that the research was conducted in the absence of any commercial or financial relationships that could be construed as a potential conflict of interest.

## Supplementary material

Supplementary Figure S1 **BiFC positive interaction control and auto-fluorescence test. (A)** *N. benthamiana* leaves were agro-infiltrated with pYFN43-AKIN10 and pYFC43-AKINβ2 vectors. The fluorescence was analyzed by laser-scanning confocal microscopy. From left to right: the GFP and DAPI fluorescence spectrum, bright field, chlorophyll fluorescence and overlay signals. **(B)** The pYFN43-OpsDHN1 and pYFN43-OpsDHN1-ΔHis vectors were transiently expressed separately and with pYFC43-AKINβ2 vector in *N. benthamiana* by agro-infiltration and analyzed with a laser-scanning confocal fluorescence microscope. The GFP fluorescence spectrum and bright field are shown. The scale bar corresponds to 50 μm.

Supplementary Figure S2 **Fluorescent visualization of (A,B) GFP::OpsDHN1-ΔSer, (C) GFP::OpsDHN1, (D) GFP::OpsDHN1-ΔHis translational fusions in *N. benthamiana* leaves**. White arrowheads indicate cytosol and nuclear signals. The fluorescence was examined by laser-scanning confocal microscopy. From left to right: the GFP and DAPI fluorescence spectrum, bright field, chlorophyll fluorescence and overlay signals. The scale bar corresponds to 10 μm.
