# Supplementary figures and images for: Nuclear localization of the dehydrin OpsDHN1 is determined by histidine-rich motif
Source: Front Plant Sci. 2015 Sep 7;6:702. doi: 10.3389/fpls.2015.00702 (PMC4561349; doi:10.3389/fpls.2015.00702)

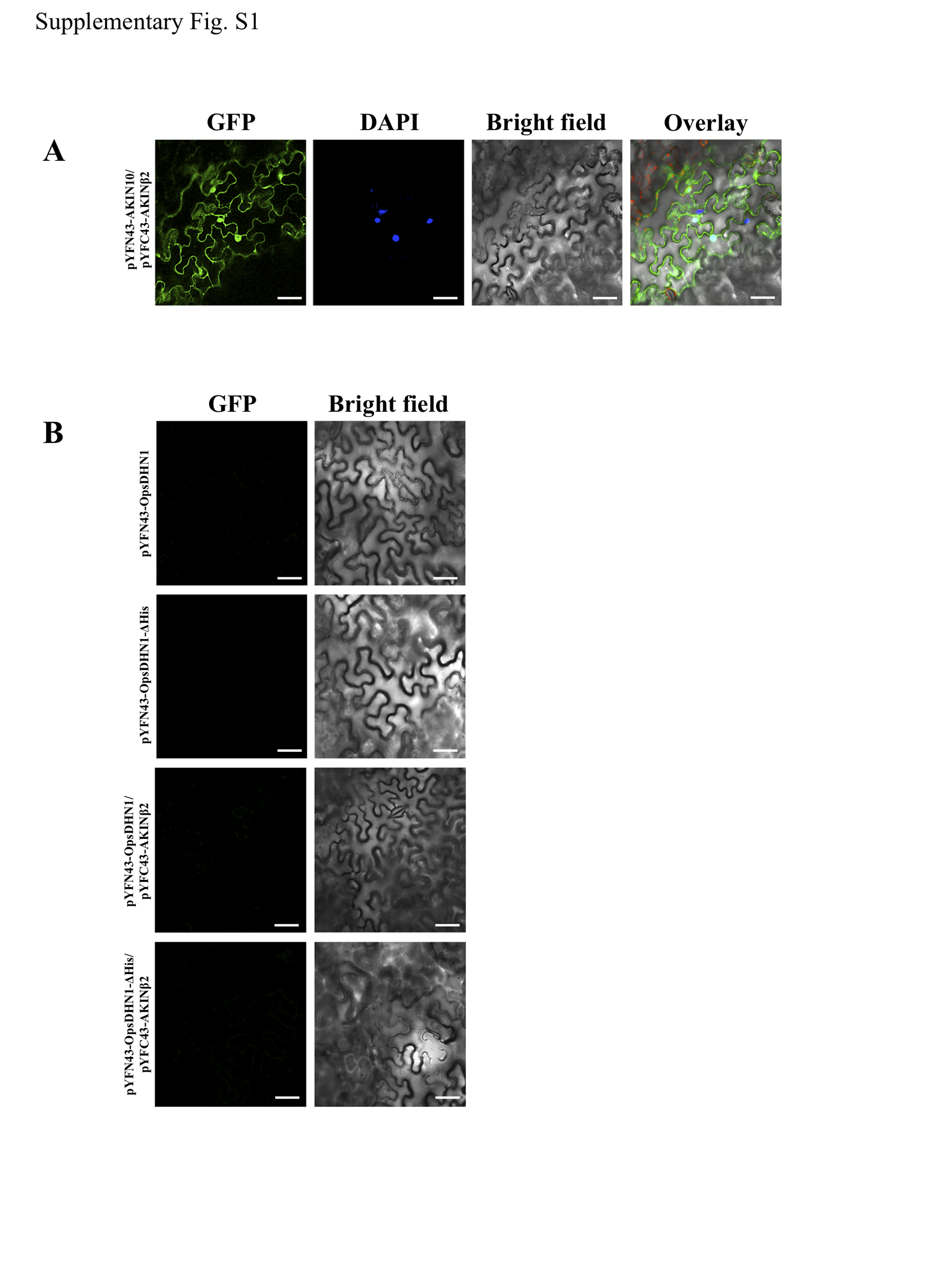

Supplement: Supplementary Figure S1 — BiFC positive interaction control and auto-fluorescence test. (A) N. benthamiana leaves were agro-infiltrated with pYFN43-AKIN10 and pYFC43-AKINβ2 vectors. The fluorescence was analyzed by laser-scanning confocal microscopy. From left to right: the GFP and DAPI fluorescence spectrum, bright field, chlorophyll fluorescence and overlay signals. (B) The pYFN43-OpsDHN1 and pYFN43-OpsDHN1-ΔHis vectors were transiently expressed separately and with pYFC43-AKINβ2 vector in N. benthamiana by agro-infiltration and analyzed with a laser-scanning confocal fluorescence microscope. The GFP fluorescence spectrum and bright field are shown. The scale bar corresponds to 50 μm. [file Image1.TIFF]

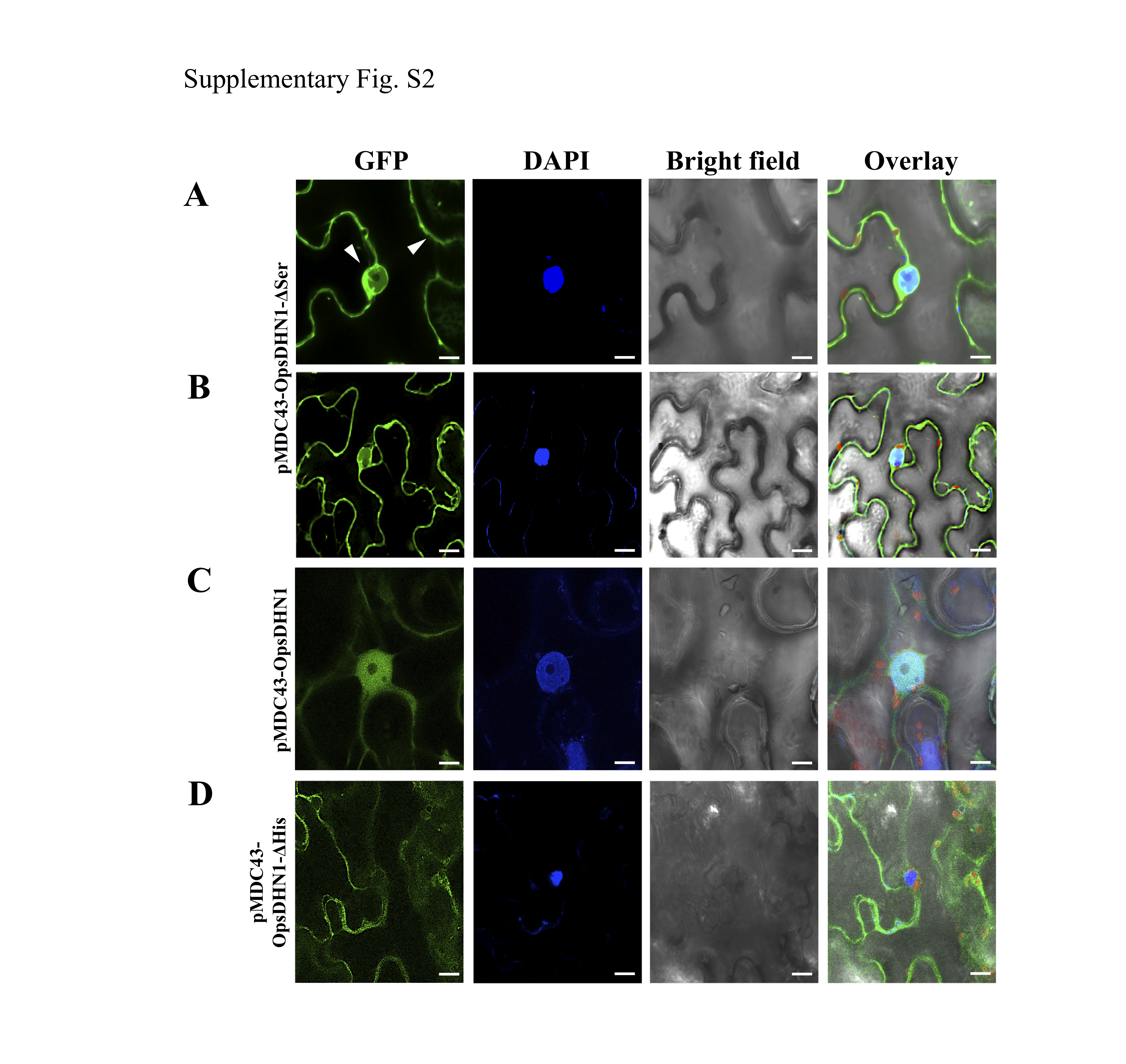

Supplement: Supplementary Figure S2 — Fluorescent visualization of (A,B) GFP::OpsDHN1-ΔSer, (C) GFP::OpsDHN1, (D) GFP::OpsDHN1-ΔHis translational fusions in N. benthamiana leaves. White arrowheads indicate cytosol and nuclear signals. The fluorescence was examined by laser-scanning confocal microscopy. From left to right: the GFP and DAPI fluorescence spectrum, bright field, chlorophyll fluorescence and overlay signals. The scale bar corresponds to 10 μm. [file Image2.TIFF]
